# Extraction, purification, in vitro antioxidant and cytoprotective ability of oligostilbenes from paeonia seeds threshing residues

**DOI:** 10.1371/journal.pone.0325485

**Published:** 2025-06-11

**Authors:** Xiao-jun Li, Heng-hui Zhang, Yong-ping Xu, Shu-ying Li, Na Li, Qing-ye Liu, Fang Zhang

**Affiliations:** 1 School of Chemistry and Chemical Engineering, North University of China, Taiyuan, China; 2 School of Bioengineering, Dalian University of Technology, Dalian, China; 3 SEM Bio-Engineering Technology Co. Ltd., Dalian, China; 4 School of Biological Science and Technology, Taiyuan Normal University, Jinzhong, China; 5 Core Laboratory, Shanxi Provincial People’s Hospital (Fifth Hospital) of Shanxi Medical University, Taiyuan, China; Lahore College for Women University, PAKISTAN

## Abstract

**Objective:**

Oligostilbenes, which have been associated with multiple biological activities, are a kind of oligomeric resveratrol compound and widely exist in Paeonia seeds threshing residues. The re-use of the Paeonia seeds threshing residues as value-added materials is, not only cost-effective, but also environmentally beneficial. It is therefore important to develop a high-efficiency method for extraction of oligostilbenes.

**Methods:**

In this investigation, different extraction methods (soxhlet extraction, high temperature and pressure extraction, cold soaking extraction, heat reflux extraction, and ultrasonic extraction) were used to extract oligostilbenes from Paeonia seeds threshing residues. By comparing the extraction yield and in vitro antioxidant ability of oligostilbenes obtained from different extraction ways, the optimal extraction technology of Paeonia seeds threshing residues oligostilbenes was selected. The macroporous resin was used to purify oligostilbenes crude extract samples, and the purification conditions were determined. The protective effect of purified oligostilbenes on oxidative damage of MODE-K cells was evaluated.

**Results:**

Ultrasonic extraction with ethanol (UA-E) possessed the highest extraction yield of oligostilbenes, and the extraction yield was (3.45 ± 0.07)%. The oligostilbenes extracts obtained by different extraction methods had scavenging ability on DPPH· and ABTS^+^·, and UA-E showed relatively stronger scavenging ability at different concentration levels. The best resin for purifying oligostilbenes was X-5, and the adsorption and desorption rates were (93.12 ± 0.16)% and (91.33 ± 0.40)%, respectively. The optimal adsorption/desorption conditions were sample loading rate of 2 BV/h, ethanol concentration of 70%, and elution flow rate of 1.0 BV/h. There was a dose-response relationship between the scavenging ability of purified oligostilbenes on DPPH· and ABTS^+^· and the concentration of the samples. The oligostilbenes could relieve the oxidation effect of hydrogen peroxide (H_2_O_2_) on MODE-K cells, and enhance the protection of MODE-K cells by regulating the relative SOD activity, MDA, and ROS production.

**Conclusion:**

This research lays a theoretical foundation and scientific reference for the extraction, purification and application of Paeonia seed threshing residues in food and medicine.

## Introduction

Paeonia (*Paeonia suffruticosa Andrew*) is a perennial deciduous shrub of the dicotyledonous plant family Peoniaceae [1,2], which is famous as “the King of flowers” and the national flower of China [3]. Paeonia is full of treasure and has been cultivated in China for nearly 2000 years [4]. The root bark of Paeonia is well-known as a kind of traditional Chinese medicine - “Danpi”, and has been widely used as anti-cancer, anti-aggregation, anti-coagulation, and anti-inflammatory [5–7]. Paeonia flowers, stem and leaf have been proven to contain a variety of active ingredients, such as essential oil, monoterpene glycosides, flavonoids, and phenols [6,8,9]. Paeonia seeds are are rich of vegetable oil [10–12]. At present, the processing of Paeonia seeds has introduced a threshing process, which has dramatically improved the quality of its oils, but also produced more wastes such as Paeonia seeds hull. It is an important problem for the industry to make Paeonia seeds threshing residues harmless and value-added use. Reusing of the Paeonia seeds threshing residues as value-added materials is not only cost-saving, but also environmentally beneficial. Studies have revealed that Paeonia seeds and their threshing residues were rich in oligomeric compounds which have been associated with multiple biological activities [7,13].

Oligostilbenes, which are an important class of polyphenols, are oligomeric resveratrol compounds and synthesized in natural plants by the oligomerization or isomerization of resveratrol [14,15]. Studies revealed that oligostilbenes were found in Chinese herbs, such as *Astragalus*, *Codonopsis*, *Notoginseng*, Paeonia and so on [14,16], and have a variety of pharmacological activities, including antioxidant, anti-inflammatory, anti-tumor, and immunomodulatory effects. Due to its small molecular weight, better solubility and bioavailability, oligostilbenes have attracted much attention in drug research. Tie et al [17] isolated four new oligostilbenes, and confirmed anti-inflammatory effects of Iris *lactea* oligostilbenes in LPS-induced RAW 264.7 cells. Oligostilbenes of Paeonia *lactiflora* seeds could be potent GLP-1 secretagogue targeting the TGR5 receptor [18], and exhibited significant cytotoxic activity against human cancer cell lines (Hela and MCF-7) [19]. He et al [20] isolated ten oligostilbenes from Paeonia suffruticosa seed extract, and confirmed 1 μM oligostilbenes all demonstrated anti-apoptotic effects on rabbit OA chondrocytes. Studies revealed that oligostilbenes purified from Paeonia *suffruticosa* could inhibit the proliferation of cancer cells by reducing lactic acid synthesis and TXNIP expression [21]. So far, fewer reports have focused on the high-efficiency extraction and enrichment of oligostilbenes owing to the relatively low content in plants. Therefore, it is very important to select a suitable extraction method for oligostilbenes.

Various extraction technologies are crucial for isolating active ingredients from plant matrices. These methods include cold dip extraction, heat reflux extraction, ultrasound or microwave-enhanced isolation, and extraction using supercritical fluids. No matter which extraction method is used, the ultimate goal is to improve the extraction efficiency on the premise of maintaining the molecular structures and biological activity of the extract. Oligostilbenes molecules are rich in hydroxyl groups, and changes in extraction temperature, solution polarity, pH value and so on could result in the instability of oligostilbenes [22,23].

This study focused on comparing 8 different extraction technologies of oligostilbenes and their antioxidant activities, and then identifying a proven and effective method for the extraction of total oligostilbenes from Paeonia seeds threshing residues. In addition, the extracts were purified by macroporous resins. DPPH· and ABTS^+^· scavenging ability of oligostilbenes before and after purification were evaluated. A H_2_O_2_-induced oxidative damage model of MODE-K cells was established to evaluate the oxidation resistance ability, and the level of SOD, MDA, and ROS were examined to evaluate the protective effect of purified oligostilbenes on oxidative damage of MODE-K cells.

## Materials and methods

### Materials and reagents

Paeonia seeds threshing residues were collected in October 2023 from Shennong Zhihua Biotechnology (Shanxi) Co., Ltd. County located in Changzhi, Shanxi Province, China. The threshing residues were air-dried and stored at −18°C.

2,2-diphenyl-1-picrylhydrazyl (DPPH), 2,2’-azinobis-(3-ethylbenzothiazoline-6-sulfonic acid ammonium salt (ABTS), folin-Ciocalteu (F-C) reagent, gallic acid, rutin and vitamin C (Vc) were purchased from Sigma Chemical Co. (St. Louis, MO, USA). Analytical-grade methanol, ethanol, Na_2_CO_3_, and K_2_S_2_O_8_ were purchased from Tianjin Beichen District Fangzheng Reagent Factor (Tianjin, China). D101, D201, NKA, HP-20, and X-5 macroporous resins were purchased from Beijing Solarbio Science & Technology Co., Ltd. (Beijing, China).

### Oligostilbenes extraction by different methods

Soxhlet extraction (SE): 10 g of Paeonia seeds threshing residues powder wrapped with filter paper was introduced in a thimble fixed over a distillation flask, and 100 mL 95% ethanol solution in the flask was heated to 100°C for 4 h. The ethanol vapor got condensed into liquid and dripped into the condenser, the extraction of oligostilbenes from Paeonia seeds threshing residues occurred [24]. This process was performed in the SEM-06 Soxhlet extractor (Shanghai Benang Scientific Instrument Co., LTD).

High temperature and pressure extraction (HTP): 10 g of Paeonia seeds threshing residues powder was included in deionized water [solid-liquid ratio: 1:10 (g/mL)]. The mixture was placed in a DGL-75GI pressure steam sterilizer (Shanghai Braun Co. LTD) at 121°C, 100 kPa for 30 min.

Cold soaking extraction (CE): 10 g of Paeonia seeds threshing residues powder was included in 95% ethanol solution (CE-E) or deionized water (CE-W) [solid-liquid ratio: 1:10 (g/mL)]. The mixture was soaked for 24 h at room temperature.

Heat reflux extraction (HR): 10 g of Paeonia seeds threshing residues powder was included in 95% ethanol solution (HR-E) or deionized water (HR-W) [solid-liquid ratio: 1:10 (g/mL)]. The mixture was subjected to the heat reflux cycles at the temperature of 80°C for 80 min [25].

Ultrasound-assisted extraction (UA): 10 g of Paeonia seeds threshing residues powder was included in 95% ethanol solution (UA-E) or deionized water (UA-W) [solid-liquid ratio: 1:10 (g/mL)]. The mixture was subjected to an ultrasound system (40 kHz, 120 W) at 40°C for 40 min [26].

All the extracted solution was centrifuged at 5000 × g for 15 min (TDL-60B, Anting, Shanghai, China), and the supernatant was freeze-dried and stored at −18 °C before analysis.

### Determination of the oligostilbenes

The oligostilbenes are a class of polyphenol compound, and its determination is usually based on polyphenol analysis techniques which is according to the method of Zhang et al with minor modifications [22]. Briefly, 0.1mL of each sample solution was mixed with 6 mL of distilled water, and then 0.5 mL of F-C reagent, 1.5 mL of 20% (w/v) Na_2_CO_3_ solution, and 1.9 mL of distilled water were added in order. The mixture solution was kept at 20°C for 30 min, and the absorbance was measured at 750 nm with a WFG-7200 spectrophotometer (INESA Analytical Instrument Co., Ltd., Shanghai, China). Its results were expressed as a gallic acid equivalent percentage of crude extract (%), gallic acid standard curve is as follow, *Y* = 1.0353*X* + 0.0052 (*R*^*2*^ = 0.9992).

### Resin pretreatment

The macroporous resin was firstly pretreated with 90% ethanol, 5% hydrochloric acid solution, 5% NaOH solution, and deionized water in sequence to remove the impurity according to the method of Duan, et al [27].

### Static adsorption/desorption of oligostilbenes

5 pretreated macroporous resins, D101, D201, NKA, HP-20 and X-5, were vacuumized to remove excess water. 2 g (W) resins were weighed into a 50 mL conical flask, and 25 mL (V_1_) of the sample solution (C_0_) was added. The conical flask was placed in an oscillating chamber and shaken at room temperature for 24 h. 1 mL of supernatant was taken to determine the mass concentration of oligostilbenes (C_1_). A little distilled water was used to rinse the residual crude extract on the surface of various resins that were fully adsorbed, and then vacuumed filtrated. After removing the water, 25 mL of 50% ethanol solution (V_2_) was added to desorption for 24 h, and 1 mL of desorption solution was taken to determine the mass concentration of Oligostilbenes of Paeonia seeds threshing residues. The adsorption capacity (Q), adsorption rate (A), and desorption rate (P) were calculated according to the formula below [28], and the best suitable resin for purification of oligostilbenes from Paeonia seeds threshing residues was screened.


Q = (C0−C1× V1/W (1)

A (%) = [(C0−C1)/C0] ×100 (2)

P (%) = (V2×C)/(W×Q) ×100 (3)

### Dynamic column chromatography of oligostilbenes

30 g pre-treated X-5 resin was accurately weighed and loaded into a 2.5 cm × 60 cm glass column for dynamic adsorption/desorption processes. The bed volume was fixed at 60 BV, after equilibrium for 12 h, sample concentration (1 mg/mL, 2 mg/mL, 4 mg/mL, 6 mg/mL), sample loading rate (2 BV/h, 3 BV/h, 4 BV/h, 5 BV/h), eluent (ethanol) dosage (50%, 60%, 70%) and elution flow rate (1 BV/h, 2 BV/h, 3 BV/h, 4 BV/h) were investigated respectively. The desorption capacity was calculated based on the oligostilbenes content.

### Assessment of in vitro antioxidant activity

DPPH· radical scavenging activity was evaluated by the modified procedures of Choi et al [29]. Different concentrations were prepared from Paeonia seeds threshing residue extract using the serial dilution technique with ethanol solvent. Briefly, 0.3 mL of diluted extract and 2.7 mL of 60 μM DPPH in ethanol were mixed, and the mixture reacted at 25°C for 30 min in the dark and absorbance value (As) at 517 nm was determined by using UV-visible spectroscopy. The absorbance value (OD) of the reaction liquid (0.3 mL distilled water and 2.7 mL DPPH) was recorded as A_0_, and the OD value of the reaction solution (0.3 mL sample solution with different concentrations and 2.7mL anhydrous ethanol) was recorded as Ar. Vc was used as the positive control. Finally, the scavenging potential of extract samples was determined by the formula below:


DPPH· scavenging activity (%) = [1−(As – Ar)/A0] × 100 (4)

The ABTS^+^· scavenging assay was come into effect by a reported procedure with some modifications [30]. ABTS working solution were prepared as follow: 7 mmol/L ABTS solution and 2.5 mmol/L potassium persulfate solution were mixed in the equal volume and reacted for 12 ~ 16 h, then the mixed solution was diluted with 80% ethanol until its absorbance value was 0.7 at 734 nm. 0.3 mL of oligostilbenes sample and 2.7 mL of ABTS working solution was mixed for 30 min, and the absorbance value was measured at 734 nm, the results were expressed as As. The absorbance value of the mixture of 0.3 mL distilled water and 2.7 mL ABTS working solution was recorded as A_0_. Vc was used as a positive control. The scavenging rate of ABTS^ +^ · was calculated according to formula 5.


ABTS+· scavenging activity =(A0−As)/A0× 100 (5)

### Construction of cell oxidative damage model

The oxidative damage induced by H_2_O_2_ was carried out using the described methods with some modifications [31,32]. MODE-K cells were grown in 1640 complete medium enhanced with 10% FBS and kept at 37◦C within a specific environmental setting comprising 5% CO_2_. These cells, with a concentration of 1 × 10^4^ cells per well, were placed in 96-well plates and permitted to cultivate for 24 h. Then different concentrations of H_2_O_2_ (0 μM, 100 μM, 200 μM, 300 μM, 400 μM, 500 μM) were added to incubate for 4 h. The toxic effects of the specimens on MODE-K cells were evaluated through a CCK-8 assay kit (Beyotime, China)

### Toxicity of oligostilbenes on MODE-K

MODE-K cells were inoculated into 96-well plates. After the cells were attached to the wall, the control group and the experimental group were set up (oligostilbenes concentration: 12.5 μg/mL, 25 μg/mL, 50 μg/mL, 100 μg/mL, 200 μg/mL, 400 μg/mL,). The cell survival rate was determined using a CCK-8 assay kit.

### Toxicity of oligostilbenes on MODE-K cells damaged by H_2_O_2_

MODE-K cells were inoculated into 6-well plates for 24 h. The experimental group was pretreated with oligostilbenes of 25 mg/L, 50 mg/L and 100 mg/L in each well, and the control group and model group were pretreated with the same amounts of complete media, respectively for another 24 h. Then the model group and the experimental group were added with an equal amount of 300 μM H_2_O_2_, and continued to be cultured for 4 h, the cell survival rate was determined by the CCK-8 method.

### Antioxidant effects of oligostilbenes on MODE-K cells

MODE-K cells were cultured in 1640 complete medium supplemented with 25 μg/mL, 50 μg/mL, and 100 μg/mL of purified oligostilbenes for a period of 24 h. Post this incubation, the medium was discarded, and solutions of 300 μM H_2_O_2_ were introduced to the cells in a 6-well plate. For control purposes, wells with no oligostilbenes (serving as a control group) and wells without H_2_O_2_ (acting as a model group) were also prepared. After an additional 4 h of incubation, the cells were washed twice with PBS and then collected, the intracellular SOD, MDA and ROS levels were measured with the SOD, MDA and ROS assay kits from Beyotime Biotechnology, Shanghai, China, and the total protein content of each sample was determined by BCA Protein Assay Kit [33].

### Statistical analysis

The experiment was repeated three times and all data were presented as mean ± SD (standard deviation). Statistical analysis was done with one-way ANOVA using SPSS 22.0 (IBM Crop. Armonk, NY, USA). *p* < 0.05 was considered to be statistically significant.

## Results and discussion

### Oligostilbenes extraction by different methods

**Fig 1:**
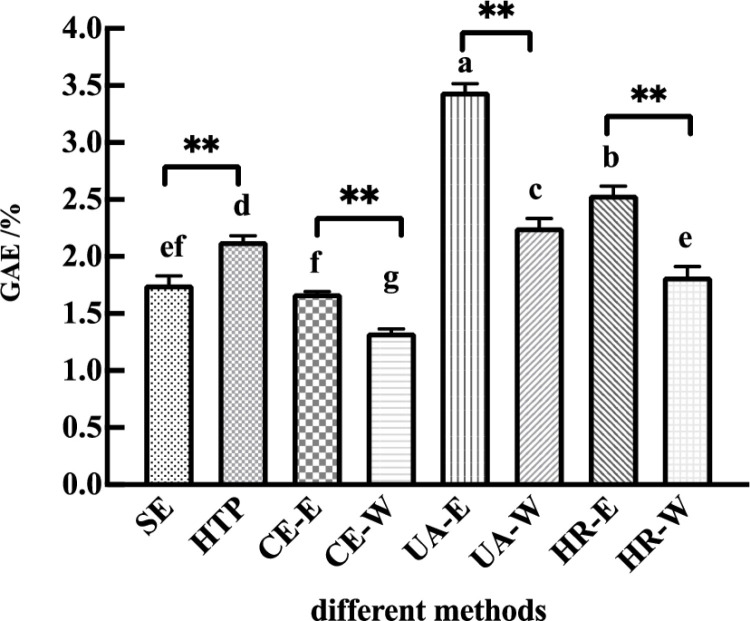
Effects of different extraction methods on the yield of oligostilbenes. Data were presented as means ± SD (n = 3). Different lowercase letters of a, b, c, d, e, and f represent significant differences (*p* < 0.05). “**” represent significant differences (*p* < 0.01).

Different extraction methods had significant effects on the extraction yield of oligostilbenes (*p* < 0.05). As can be seen from Fig 1, the extraction solvent, extraction temperature, extraction pressure and ultrasonic assistance had significant effects on the extraction yield of oligostilbenes from Paeonia seeds threshing residues. The extraction yield of oligostilbenes with ethanol as solvent was significantly higher than that with pure water as solvent (CE-E > CE-W, HR-E > HR-W, UA-W > UA-E), which was because that ethanol had highlighted the selectivity of oligostilbenes in the solid-liquid extraction process. In addition, high temperature and high pressure are also conductive to the migration of oligostilbenes (the solid solute) to ethanol (the liquid solvent) [34]. In the ultrasonic extraction method, due to the auxiliary ultrasonic cavitation and ultrasonic crushing effect, UA-E obtained the highest yield of oligostilbenes ((3.45 ± 0.07) %) among the 8 extraction methods.

### Static adsorption/desorption of oligostilbenes

**Fig 2:**
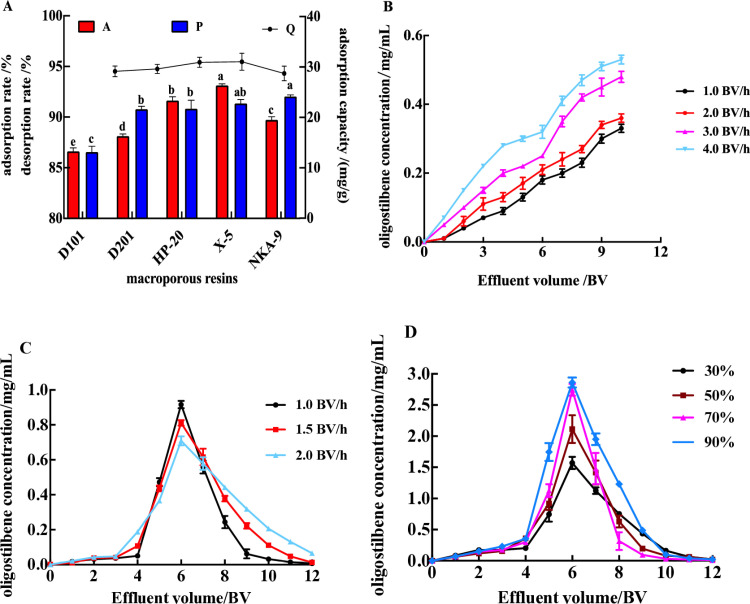
Purification results of oligostilbene. (A) Effects of different macroporous resins on the adsorption capacity, adsorption rate, and desorption rates of oligostilbene. Different lowercase letters of a, b, c, d, e, and f represent significant differences (p < 0.05); (B) Effects of sample loading rate on adsorption/desorption ratio of X-5 resin; (C) Effects of eluent (ethanol) concentration on adsorption/desorption ratio of X-5 resin; (D) Effects of elution flow rate on adsorption/desorption ratio of X-5 resin.

The adsorption/desorption ability of macroporous resins to target compounds is the key evaluation index for selecting resins [35]. The stronger of this ability, the higher of the utilization rate of the resin, and the purification efficiency of the target substance. The physical properties of 5 resins (D101, HP-20, X-5, D201, NKA-9) are listed in Table 1. The 5 kinds of macroporous resins selected in the experiment have different characteristics, such as polarity (D101 and HP-20 of non-polar, X-5 of weak-polar, D201and NKA-9 of strong polar), surface Area (D101 > HP-20 > X-5 > D201 > NKA-9), and average pore diameter (X-5 > NKA-9 > D201 > HP-20 > D101), which may influence the adsorption/desorption ability in the purification stage [36]. As shown in Fig 2 (A), X-5 displayed the uppermost adsorption capacity ((31.02 ± 1.69) mg/g) among the five types of resins, but there was no statistically significant difference. However, the adsorption rate of X-5 ((93.12 ± 0.16)%) was significantly higher than HP-20 ((91.62 ± 0.39)%), NKA-9 ((89.72 ± 0.31)%), D201 ((88.13 ± 0.21)%) and D101 ((86.62 ± 0.33%), X-5 was a kind of weak-polar resin and possesses a larger surface area and average pore diameter. In addition, NAK-9 and X-5 showed the highest desorption rate ((92.04 ± 0.16)% and ((91.33 ± 0.40)%) among five macroporous resins, followed by HP-20 ((90.82 + 0.85)%), D201 ((90.76 + 0.32)%) and D101 ((86.55 ± 0.58)%). X-5 exhibits the best adsorption/desorption properties and therefore was chosen as the best macroporous resin for the following purification of oligostilbenes from Paeonia seeds threshing residues [37].

**Table 1:** Physical parameters of five resin.

Resin	Particle Size (mm)	Average Pore Diameter (nm)	Surface Area (m^2^/g)	Polarity
D101	0.3 ~ 1.25	0.3 ~ 1.2	600 ~ 700	Non-polar
HP-20	0.3 ~ 1.25	10.0 ~ 12.0	500 ~ 550	Non-polar
X-5	0.3 ~ 1.25	21.0 ~ 23.0	500 ~ 600	Weak-polar
D201	0.3 ~ 1.25	10.0 ~ 13.0	200 ~ 300	polar
NKA-9	0.3 ~ 1.25	15.5 ~ 16.5	170 ~ 250	polar

### Effects of sample loading rate on adsorption/desorption ratio of X-5 resin

The sample loading rate had a great influence on the concentration of oligostilbenes in effluent Fig 2(B). With the increase of loading rate, the concentration of oligostilbenes increased, which indicated that the adsorption capacity of X-5 resin decreased, and the earlier the leakage point (the concentration of oligostilbenes in the effluent is 20% of the initial concentration) appeared, the less the crude extract was treated. When the sample flow rate was 3.0 BV/h and 4.0 BV/h, the oligostilbenes compounds could not be fully absorbed by macroporous resin due to the large flow rate, and the oligostilbenes compounds began to leak prematurely. At 1.0 BV/h and 2.0 BV/h, oligostilbenes could fully diffuse in the resin column, and the adsorption capacity of the resin was larger, and the leakage point only appeared when the crude extraction liquid volume of oligostilbenes was 9 BV and 8 BV, respectively. However, the sample loading rate of 1.0 BV/h was too slow, which affected the purification efficiency. Therefore, the optimal sample loading rate was 2 BV/h in this experiment.

### Effects of elution flow rate on adsorption/desorption ratio of X-5 resin

The effect of elution flow rate on the enrichment and purification of oligostilbenes was significant (Fig 2(C)). With the increase of the eluent flow rate (1.0 BV/h, 1.5 BV/h and 2.0 BV/h), the worse the eluent performance, the more serious the tailing phenomenon, the more eluent needed, and the lower the content of oligostilbenes compounds in the eluent. This may be due to that, the eluent and oligostilbenes compounds absorbed during the absorption stage could not fully contact with the excessive elution flow rate, resulting in oligostilbenes compounds could not be fully dissolved in the eluent. When the elution flow rate was 1.0 BV/h, not only the peak height was higher, but also the peak shape was narrow and the convergence was better, which was conducive to the enrichment and purification of oligostilbenes compounds. Therefore, the elution flow rate of 1.0 BV/h was selected.

### Effects of eluentconcentration on adsorption/desorption ratio of X-5 resin

It can be seen from Fig 2(D) that the concentration of ethanol had a great influence on the desorption of oligostilbenes. With the increase of elution concentration (30%−70%), the absolute convergence of the desorption curve was enhanced, the peak shape was narrowed, and the tailing phenomenon was weakened, so that the oligostilbenes compounds could be better enriched. However, 90% ethanol concentration not only had a lower desorption peak, but also had a wider peak shape and a serious tailing phenomenon, which was not conducive to the enrichment and purification of oligostilbenes compounds. In consideration of the production cost and efficiency, 70% ethanol was determined as the best elution solvent. Under the condition of 70% ethanol as eluent and the elution volume of 12 BV, the oligostilbenes concentration after purification was 118.44 mg/g, which was 3/1 of the crude extract.

### DPPH· and ABTS + · scavenging ability of oligostilbenes before purification

Excessive free radicals (DPPH·, ABTS^+^·, ·O^2-^ and so on) were unsaturated electronic substances, which can compete for electrons with protein in the human body. Excessive free radicals could cause oxidative stress and induce many human diseases, such as cancer, senescence, inflammation, and cardiovascular disease [20]. In this study, with Vc as the positive control, the DPPH· and ABTS^+^· scavenging assays were carried out to compare the antioxidant activity of oligostilbenes samples obtained by eight different extraction processes. As shown in Table 2, within the range of experimental concentration (50–400 ug/mL), the DPPH· scavenging ability of different samples increases gradually with the increase of oligostilbenes concentration. At the same concentration level, Vc showed a significant DPPH· scavenging ability compared with the other 8 samples. The DPPH· scavenging ability of oligostilbenes samples obtained by ethanol extraction method (SE, CE-E, HR-E, and UA-E), was stronger than that of oligostilbenes samples obtained by other 4 kinds of pure water extraction (CE-W, HR-W, UA-W, and HPT), in which the scavenging ability of oligostilbenes samples obtained by SE (50 ug/mL, 200 ug/mL, and 400 ug/mL) and CE-E (300 ug/mL) was the weakest. In addition, at the concentration level of 100 ug/mL, the oligostilbenes samples obtained by CE-W, HR-W, UA-W were also weakest, and there was no significant difference (*p* > 0.05). The reason may be due to the high temperature caused damage to oligostilbenes active center, and some chemical structures with antioxidant effects cannot be dissolved in water. As can be seen from Table 3, with the increase of Vc and oligostilbenes concentration, the ABTS + · scavenging rate increased continuously, and there was a dose-effect positive correlation between the ABTS + · scavenging rate of different samples and the sample concentration. At different concentration levels (50–400 ug/mL), Vc showed the strongest ABTS^+^· scavenging ability, followed by oligostilbenes obtained by SE and UA-E. Similar to the results of DPPH· scavenging ability, oligostilbenes samples with ethanol as extraction solvent (SE, CE-E, HR-E and UA-E) had a stronger scavenging effect on ABTS^+^· than those with pure water as extraction solvent (CE-W, HR-W, UA-W, and HTP), and there were significant differences. According to the above findings, oligostilbenes extracted by eight methods demonstrated different antioxidant abilities. All oligostilbenes samples have antioxidant abilities and could be potential sources of bioactive ingredients, especially oligostilbenes obtained UA-E, which was consistent with the results of oligostilbenes extraction by different methods.

**Table 2:** DPPH· radical scavenging activity.

Conc. (ug/mL)	DPPH· radical scavenging activity of oligostilbenes extraction by different methods (%)								
	Vc	SE	HPT	CE-E	CE-W	HR-E	HR-W	UA-E	UA-W
50	90.75 ± 0.68 a	10.74 ± 0.90 d	10.79 ± 1.15 d	13.6 ± 0.71 c	10.34 ± 0.60 d	13.29 ± 0.61 c	10.17 ± 0.27 d	15.31 ± 0.45 b	11.40 ± 0.56 d
100	93.44 ± 0.76 a	15.74 ± 1.04 c	15.83 ± 0.31 c	15.62 ± 1.18 cd	13.86 ± 0.38 de	17.74 ± 0.66 b	14.52 ± 0.59 cde	17.59 ± 0.47 b	14.37 ± 0.55 de
200	98.63 ± 1.03 a	17.05 ± 0.62 f	16.80 ± 0.10 f	16.47 ± 0.48 f	18.28 ± 0.59 e	23.42 ± 0.57 b	21.75 ± 0.76 c	19.39 ± 0.5 d	18.88 ± 0.24 de
300	92.84 ± 1.06 a	20.69 ± 1.06 d	20.85 ± 0.96 d	18.37 ± 0.91 e	25.20 ± 0.42 c	26.63 ± 0.47 b	25.34 ± 0.55 bc	25.65 ± 0.78 bc	20.82 ± 0.42 d
400	98.69 ± 0.83 a	21.4 ± 0.62 f	21.47 ± 0.59 f	21.53 ± 1.06 f	28.63 ± 0.57 d	31.42 ± 0.63 c	29.72 ± 0.54 d	37.60 ± 0.56 b	25.25 ± 0.35 e

* Values followed by the same small letter within the same line are not significantly different (p > 0.05) according to Duncan multiple range test.

**Table 3:** ABTS^+^· radical scavenging activity.

Conc. (ug/mL)	DPPH· radical scavenging activity of oligostilbenes extraction by different methods (%)								
	Vc	SE	HPT	CE-E	CE-W	HR-E	HR-W	UA-E	UA-W
50	90.75 ± 0.68 a	10.74 ± 0.90 d	10.79 ± 1.15 d	13.6 ± 0.71 c	10.34 ± 0.60 d	13.29 ± 0.61 c	10.17 ± 0.27 d	15.31 ± 0.45 b	11.40 ± 0.56 d
100	93.44 ± 0.76 a	15.74 ± 1.04 c	15.83 ± 0.31 c	15.62 ± 1.18 cd	13.86 ± 0.38 de	17.74 ± 0.66 b	14.52 ± 0.59 cde	17.59 ± 0.47 b	14.37 ± 0.55 de
200	98.63 ± 1.03 a	17.05 ± 0.62 f	16.80 ± 0.10 f	16.47 ± 0.48 f	18.28 ± 0.59 e	23.42 ± 0.57 b	21.75 ± 0.76 c	19.39 ± 0.5 d	18.88 ± 0.24 de
300	92.84 ± 1.06 a	20.69 ± 1.06 d	20.85 ± 0.96 d	18.37 ± 0.91 e	25.20 ± 0.42 c	26.63 ± 0.47 b	25.34 ± 0.55 bc	25.65 ± 0.78 bc	20.82 ± 0.42 d
400	98.69 ± 0.83 a	21.4 ± 0.62 f	21.47 ± 0.59 f	21.53 ± 1.06 f	28.63 ± 0.57 d	31.42 ± 0.63 c	29.72 ± 0.54 d	37.60 ± 0.56 b	25.25 ± 0.35 e

* Values followed by the same small letter within the same line are not significantly different (p > 0.05) according to Duncan multiple range test.

### DPPH· and ABTS^+^· scavenging ability of oligostilbene after purification

**Fig 3:**
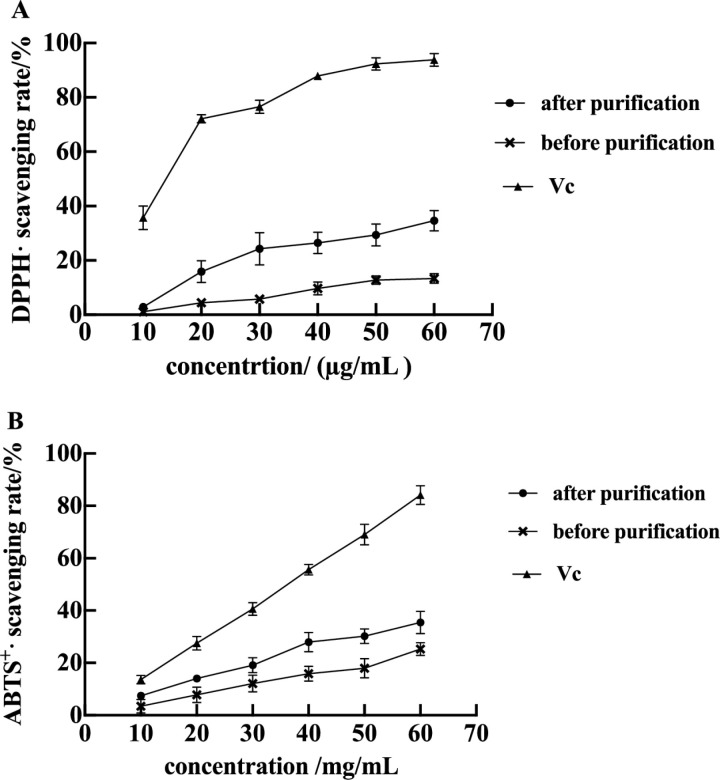
Scavenging activity of the different samples and Vc against DPPH· (A) and ABTS^+^· (B).

Paeonia seeds and its shell have been proven to be a potential source of antioxidant oligostilbenes [18,38]. The above research in this study proved the antioxidant abilities of oligostilbenes crude extract, and the in vitro antioxidant ability of purified oligostilbenes was carried out by the DPPH· and ABTS^+^· scavenging assays. The antioxidant abilities of purified oligostilbenes were determined by DPPH· and ABTS^+^· scavenging assays. The DPPH· scavenging ability of Vc, oligostilbenes extracted by UA-E before and after purification were shown in Fig 3(A). Within the experimental concentration range (10−60 ug/mL), the DPPH· scavenging rate gradually increased with the increase of sample concentration. The DPPH· scavenging ability of purified Paeonia oligostilbenes was significantly higher than that of unpurified Paeonia oligostilbenes, and significantly lower than that of Vc. These results indicated that the DPPH· scavenging ability was significantly enhanced by the above purification method, and the oligostilbenes could be significantly enriched under the selected purification conditions. The ABTS ^+^ · scavenging ability of Vc, oligostilbenes extracted by UA-E before and after purification was shown in Fig 3(B). As can be seen from the figure, with the increase of sample concentration, the ABTS^ +^ · scavenging rate of Paeonia oligostilbenes gradually increased. After purification, the ABTS^ + ^· scavenging ability of purified oligostilbenes in Paeonia seeds threshing residues was significantly higher than that of unpurified, indicating that after purification by X-5 macroporous resin, the content of oligostilbenes in Paeonia seeds threshing residues were increased, and the anti-ABTS^+^· activity was enhanced. The results were consistent with the data reported by Vania Saez et al [39] and Rodriguez-Bonilla et al [40].

### Toxicity of oligostilbenes and H_2_O_2_ on MODE-K

Oxidative stress has an important impact on animal reproduction and growth. MODE-K cells which are intestinal epithelial cells, are physical barrier between the body and intestinal flora.

Oxidative damage caused by H_2_O_2_ may lead to breakdown of this barrier and affecting health [41]. Studies have shown that oligostilbenes has anti-tumor, anti-inflammation, anti-oxidation, anti-free radical effects [17–20]. The phenolic hydroxyl group in oligostilbenes molecule makes it demonstrate strong antioxidant abilities, but oligostilbenes also have direct cytotoxic effect, so it is of great significance to understand the protective or inhibitory effect of oligostilbenes on cells under concentration conditions for the future use of oligostilbenes in some diseases related to oxidative stress, such as stroke, emphysema, Alzheimer’s disease, and diabetes.

**Fig 4:**
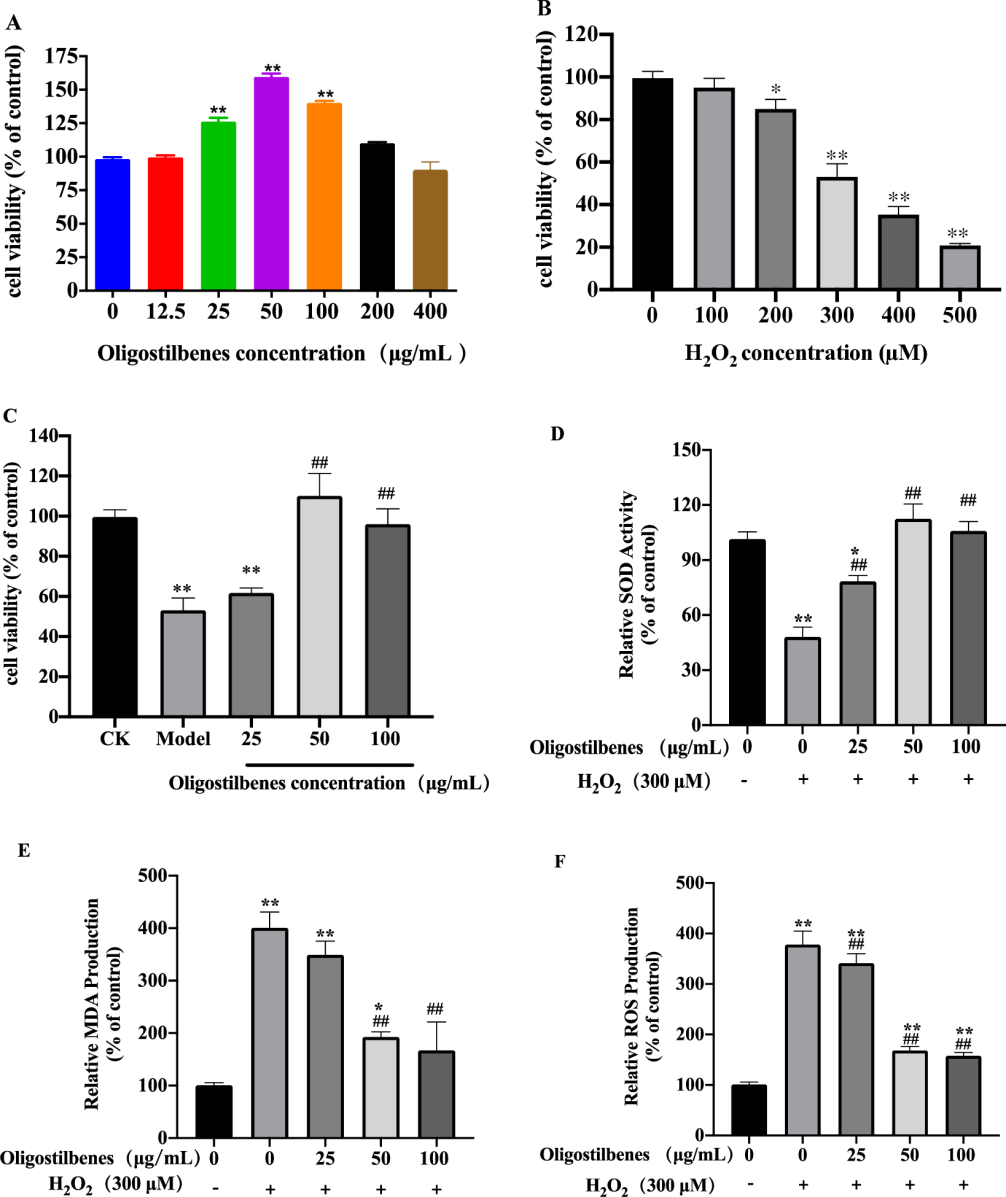
Protective effect of oligostilbenes on oxidative damage of MODE-K cells. (A) Toxicity of oligostilbenes on MODE-K, (B) Toxicity of H_2_O_2_ on MODE-K, (C) Toxicity of oligostilbenes on MODE-K cells damaged by H_2_O_2_. (D) The relative SOD activity of MODE-K cells. (E) The relative MDA production of MODE-K cells. (F) the relative MDA and ROS production of MODE-K cells. Data were presented as means ± SD (n = 3), *p < 0.05, **p < 0.01, versus control group (CK); #*p* < 0.05, ##*p* < 0.01 versus H_2_O_2_ group.

As can be seen from Fig 4(A), compared with the control group, the cell viability of MODE-K can be improved when the concentration of oligostilbenes was in the range of 0–200 μg/mL. In the range of 0–50 μg/mL, the cell viability increased with the increase of oligostilbenes concentration. When the oligostilbenes concentration was 50 μg/mL, the cell viability reached the highest (160%). In the range of 50–200 μg/mL, the cell viability began to decline with the increase of the concentration, and 400 mg/L was the lowest cell viability. Consequently, levels of 25 μg/mL (low dose), 50 μg/mL (medium dose), and 100 μg/mL (high dose) were chosen for further experiments.

The cell viability decreased with the increase of H_2_O_2_ concentration in the range of 0 ~ 500 μM (Fig 4(B)), After intervention for 4 h, when the concentration of H_2_O_2_ was greater than 200 µmol/L, the cell viability of MODE-K cells was significantly lower than that of the control group (*p* < 0.05). The cell viability of MODE-K was (53.07 ± 6.12) % with an H_2_O_2_ concentration of 300 µmol/L, and the cell viability of MODE-K was lower than (35.24 ± 3.87) % with an H_2_O_2_ concentration of more than 400 µg/mL. It can be seen that when the H_2_O_2_ concentration was 300 µmol/L, MODE-K cells could not only retain certain cell activity, but also simulate apoptosis, therefore, an H_2_O_2_ concentration of 300 µmol/L was selected for subsequent experiments.

Fig 4(C) showed the proliferation of MODE-K cells with the intervention of different concentrations of oligostilbenes (25 μg/mL, 50 μg/mL, and 100 μg/mL). The results showed that after the intervention of oligostilbenes for 24 h, the antioxidant effect of MODE-K cells rose in varying degrees and a concentration-dependent manner. Compared with the control group (without H_2_O_2_), the cell viability of MODE-K in the model group (53.07 ± 6.12) % significantly decreased with an H_2_O_2_ concentration of 300 µmol/L (*p* < 0.05). When the intervention concentration of oligostilbenes was 25 µg/mL, the cell viability of MODE-K cells was (61.68 ± 2.52) %, which was significantly different from the CK group (*p* < 0.05), and was not significantly different from the model group (*p* < 0.01). When the intervention concentration of oligostilbenes was 50 µg/mL and 100 µg/mL, the cell viability of MODE-K cells was (110.10 ± 11.18) % and (95.57 ± 7.72) % respectively, and there was a significantly difference with the model group (p < 0.01). This may be due to the fact that oligostilbenes change the sensitivity of cell membrane to oxygen free radicals and regulates some key proteins of cell proliferation. The result indicated that oligostilbenes could reduce the oxidation effect of H_2_O_2_ on MODE-K cells, and enhance the protection of MODE-K cells. The optimal antioxidant concentration was 50 µg/mL.

### The levels of SOD, MDA and ROS

Oxidative damage is an imbalanced state of oxidative-antioxidative [3,42], which can produce many oxidative intermediates and cause cell damage [43]. Cytotoxic MDA and ROS are the most immediate and typical oxidative products, and excess secretion can reflect the peroxidative damage degree of cells and tissue [44,45]. SOD is an antioxidant metal enzyme that catalyzes the dismutation of superoxide anion radicals into oxygen and hydrogen peroxide [44]. Compared with the control group, the relative MDA and ROS production were significantly increased (*p* < 0.01), while the relative SOD activity were significantly decreased (*p* < 0.01), suggesting that oxidative damage could attenuate the antioxidant capacity of MODE-K cells (Fig 4D ~ F). With the intervention of different concentrations of oligostilbenes, the relative SOD activity significantly increased compared with the H_2_O_2_ group (*p* < 0.01) and reached levels that were not significantly different from those of the control group (except for the 25 µg/mL group). In addition, the relative MDA and ROS production of the oligostilbenes intervention group significantly decreased compared with the H_2_O_2_ group (*p* < 0.01) and reached levels that were not significantly different from those of the control group. All the changes of these oxidation-related factors indicated that purified oligostilbenes from Paeonia seeds threshing residues could help MODE-K cells resist oxidative damage. However, whether there is a balance between the protective effect of oligostilbenes and the cytotoxic effect of oligostilbenes on oxidation-damaged MODE-K cells, and how it regulates some key proteins of cell proliferation and a in vivo validation remain to be explored.

## Conclusions

Oligostilbenes are a kind of oligomeric resveratrol compound and widely exist in Paeonia seeds threshing residues. The key of the present work is to select a suitable method for the separation and purification of oligostilbenes. In this work, several extraction methods such as SE, HPT, CE (CE-E and CE-W), HR (HR-E and HR-W), and UA (UA-E and UA-W) were investigated. Among these extraction methods, UA-E showed promising results with an extract yield of (3.45 ± 0.07)%. All the oligostilbenes obtained by different extraction methods exhibited scavenging ability on DPPH· and ABTS + ·, and there was a significant difference between the various samples. Oligostilbenes obtained UA-E exhibited best scavenging ability, which was consistent with the results of oligostilbenes extraction by different methods. X-5 Macroporous resin could increase the purity of oligostilbenes samples by 3 times under the condition of a sample loading rate of 2 BV/h, ethanol concentration of 70%, and elution flow rate of 1.0 BV/h. The purified oligostilbenes showed higher scavenging ability on DPPH· and ABTS^ + ^·. The oligostilbenes could reduce the oxidation effect of H_2_O_2_ on MODE-K cells, and enhance the protection of MODE-K cells by regulating the relative SOD activity, MDA and ROS production. In addition, different doses of oligostilbenes (25 µg/mL, 50 µg/mL and 100 µg/mL) could improve antioxidant capacity in varying degrees. Oligostilbenes showed a protective effect on MODE-K cells with oxidative damage, and reduced the occurrence of diseases, which is related to the deficiency of barrier function caused by oxidation damage. The results of this study provide a scientific reference for the extraction and purification process of Paeonia seed threshing residues and the application of Paeonia seed threshing residues in food and medicine.
